# Rab7 Investigation Insights into the Existence of White Spot Syndrome Virus in Crustaceans: An *In Silico* Approach

**DOI:** 10.1155/2022/3887441

**Published:** 2022-10-20

**Authors:** Mehedi Mahmudul Hasan, M. Nazmul Hoque, Md Robiul Hasan, Mohammad Asaduzzaman, Farha Matin Juliana

**Affiliations:** ^1^Department of Fisheries and Marine Science, Noakhali Science and Technology University, Noakhali 3814, Bangladesh; ^2^Department of Gynecology, Obstetrics and Reproductive Health, Bangabandhu Sheikh Mujibur Rahman Agricultural University, Gazipur 1706, Bangladesh; ^3^Department of Biochemistry and Molecular Biology, Noakhali Science and Technology University, Noakhali 3814, Bangladesh; ^4^Department of Biochemistry and Molecular Biology, Jahangirnagar University, Savar, Dhaka 1342, Bangladesh

## Abstract

In this study, previously published Rab7 sequences from National Center for Biotechnology Information (NCBI) have been investigated from chordates, mollusks, annelids, cnidarians, amphibians, priapulids, brachiopods, and arthropods including decapods and other groups. Among decapod crustacean isolates, amino acid variations were found in 13 locations. Penaeid shrimps had variations in positions 13 (I ⟶ J), 22 (T ⟶ A), 124 (G ⟶ X), and 149 (V ⟶ X) while interestingly the freshwater prawn and mitten crab both had amino acid substitutions in positions 87 (V ⟶ C) and 95 (T ⟶ S) along with the other disagreements in amino acid positions 178 (S ⟶ N), 201 (D ⟶ E), 181 (E ⟶ D), 182 (L ⟶ I), 183 (Y ⟶ G), 184 (N ⟶ H), and 198 (A ⟶ T). Among 100 isolates of Rab7 from organisms of various phyla, mutations were observed in several positions. These mutations caused variations in hydrophobicity and isoelectric point which impact the ligand-protein binding affinity. Some common mutations were found in the organisms of the same phylum and among different phyla. Homology modeling of Rab7 proteins from different organisms was done using SWISS-MODEL and validated further by developing Ramachandran plots. Protein-protein docking showed that active residues were there in the binding interfaces of Rab7 from organisms of seven different phyla and VP28 of WSSV. Similarities were observed in the Rab7-VP28 complexes in those selected organisms which differed from the Rab7-VP28 complex in the case of Penaeid shrimp. The findings of this study suggest that WSSV may exist in different marine organisms that have Rab7 protein and transmit to crustaceans like shrimps and crabs which are of commercial importance.

## 1. Introduction

White Spot Syndrome Virus (WSSV) is a major pathogen of penaeid shrimps and crabs [1]. This is substantially a larger double-stranded (ds) DNA virus that belongs to the family *Nimaviridae* and the genus *Whispovirus* [2]. The WSSV virions are ovoid to bacilliform in shape (enveloped particles with about a length of 275 nm and a width of 120 nm) containing a flagellum-like appendage at one terminal [3]. For having a wide range of hosts, including penaeid shrimps, freshwater shrimps, crabs, lobsters, and crayfishes [2], the marine ecosystem is vulnerable to the presence of WSSV. Moreover, non-decapods may also face latent infections to act as carriers with no pathological signs [4]. Penaeid shrimps and crabs like *Scylla* spp. are cultured in ghers in which the water enters from tidal water. Post-larvae of shrimps are collected from shrimp hatcheries where mother shrimps collected from the sea are used in hatching. WSSV can enter the shrimp ghers through these hatched larvae since WSSV can be transmitted vertically. Crustaceans can also be affected by WSSV horizontally by eating dead infected organisms or by the presence of WSSV infected organisms in the water body, artificial food, and contaminated water of the gher. Horizontal transmission of WSSV was confirmed in crustaceans through cohabitation trials also [5]. It is reported that crabs can stay longer with latent infection as a carrier of the virus for their high disease resistance capacity and transmit the virus to shrimps [5, 6]. 100% of shrimps are reported to die within three to ten days of infection [7]. If shrimps and crabs are cultured together in the improved traditional shrimp ghers, WSSV can be transmitted from crabs to shrimps making shrimps more vulnerable to death due to white spot disease [8].

Rab7 is a GTP-binding protein that is reported to be the receptor for WSSV's envelope protein [9]. Rab7 is found in eukaryotes and is primarily known for its function in endocytosis and for assisting the cell in the internalization of proteins or non-particulate matter [10]. Rab proteins are known to regulate fusion and vesicle budding and are placed on the surfaces of the different exocytic and endocytic compartments that are membrane-enclosed [9]. The Rab7 is connected with late endosomes and controls the transport from early to late endosomes and the fusion of late endosomes to lysosomes [11]. Numerous diseases in eukaryotes had been observed due to the mutation in the Rab GTPases and alterations of endocytosis. Mutations associated with Rab protein activation were found connected to pathogenesis-related to genetic disorders [12]. Mutations in Rab7 were observed to be connected to generating activated forms of the protein responsible for causing disease [13]. However, Rab7 as a receptor is of substantial importance to the viral ecosystem. Relevant receptors among species control the host selection of the virus, and the expression of suitable receptors in cells of the hosts is one of the determining factors that resolves the morbific results of the disease [14]. In the chordates, annelids, cnidarians, amphibians, priapulids, and arthropods including crustaceans and other groups, Rab7 is detected. WSSV is known to kill mostly penaeid shrimps and crabs of coastal aquaculture farms. WSSV's envelop protein VP28 is recognized to be engaged in the methodical infection of shrimps over binding to the receptor Rab7 of the hosts [9]. Hasan et al. reported that mutation in the VP28 of WSSV might affect the VP28-Rab7 binding affinity and the extent of the chances of binding might be site-dependent [8]. The impacts of mutations in Rab7 protein of the decapods and non-decapods may also depend on the site and amino acid. However, disease mutations were observed to target the extremely conserved residues on the surface of Rab7 and alter nucleotide exchange [13]. McCray et al. also observed mutant Rab7 to bind with same interactors as wild-type Rab7, in the case of protein-protein interactions [13]. We predict that VP28 or any complex of proteins on the viral envelope of WSSV may have the possibility to bind Rab7 of a few other organisms as well. These organisms may play a role as vectors or asymptomatic carriers. That is why, this study focused on the analysis of amino acids of available Rab7 sequences in NCBI from different organisms from terrestrial, freshwater, marine, or brackish water and the binding of immunologically important envelope protein VP28 of WSSV to Rab7 proteins.

## 2. Materials and Methods

### 2.1. Sequence Dataset

Rab7 sequences were retrieved from NCBI (http://www.ncbi.nlm.nih.gov) with a query sequence from *Penaeus monodon* [GenBank: ABB70064.1] using BLAST (Basic Local Alignment Search Tool) [15]. 100 sequences from different organisms were selected. Organisms were from the phyla Chordata, Annelida, Cnidaria, Amphibia, Priapulida, Mollusca, and Arthropoda. Sequences were selected based on cluster to cover each phylum.

### 2.2. Multiple Sequence Alignment and Analysis

All 100 sequences were aligned using Geneious Prime trial version 2020.1 (http://www.geneious.com). Eight sequences from crustaceans (decapods) of these 100 were also aligned separately. Analysis of mutations, calculation of mean isoelectric points, and hydrophobicity was performed.

### 2.3. Construction of Phylogenetic Tree

For the construction of a phylogenetic tree, UPGMA (Unweighted Pair Group Method with Arithmetic Mean) Tree Building method was used considering “Global Alignment with free end gaps,” “Blosum62 cross-matrix,” and “Jukes Cantor Genetic Distance Model” by Geneious version 2020.1. Sequences showing significant alignments from all organisms were selected for constructing phylogenetic tree [16].

### 2.4. Protein Modelling

Homology modeling of the 3D structure of WSSV receptor Rab7 was done using SWISS-MODEL (https://swissmodel.expasy.org/) by mapping residues [17]. SWISS-MODEL template repository delivers annotation of protein quaternary structure and vital ligands to build comprehensive structural models, as well as the oligomeric structure [18]. This software produces polished 3D models on the basis of sequence alignment from the appropriate templates. The obtained models were evaluated by MolProbity version 2020.1 [19] evaluating the parameters clashscore, hydrogen bonds, van der walls contacts, geometry, rotamers, C*β* deviations, and cis-peptides. For visualization of the protein, 3D structure both PyMOL and Discovery Studio 2020 were used. The Ramachandran plots were drawn to envisage the allowed regions aimed at “backbone dihedral angles Ψ” against “*φ* of amino acid residues” [20]. Moreover, using PDB files of target, the Ramachandran plot was produced to verify whether the residues were in one of the three regions-favored, allowed, and outlier using RAMPAGE [19]. PatchDock (http://bioinfo3d.cs.tau.ac.il/PatchDock/) was used to confirm the ligand-protein interaction [21]. Discovery Studio 2020 was used for further analysis of the ligand-protein interactions.

### 2.5. Protein-Protein Docking

HADDOCK 2.4 was used for protein-protein docking of Rab7 and VP28. It clustered the number of structures into fewer clusters representing the percentage of water-refined molecules [22]. On the statistics generated from the top clusters, the reliable one was chosen. The result page reports the number of clusters and for the top clusters also the related statistics (e.g., HADDOCK score, Size, RMSD, Energies, BSA, and Z-score).

## 3. Results and Discussion

### 3.1. Rab7 Annotation

Out of 100 sequences from NCBI, 8 sequences were from the decapods of Subphylum Crustacea. 92 sequences were from the isolates of chordates, annelids, cnidarians, amphibians, priapulids, and arthropods (without decapods). WSSV receptor protein Rab7 sequence [GenBank: ABB70064] was annotated to observe five GTP-binding sites (Table 1). The putative effector binding site has 9 intervals with a length of 15 amino acids. The GDI interaction site has 8 intervals with a length of 13 amino acids.

### 3.2. Mutation Analysis

**Figure 1:**
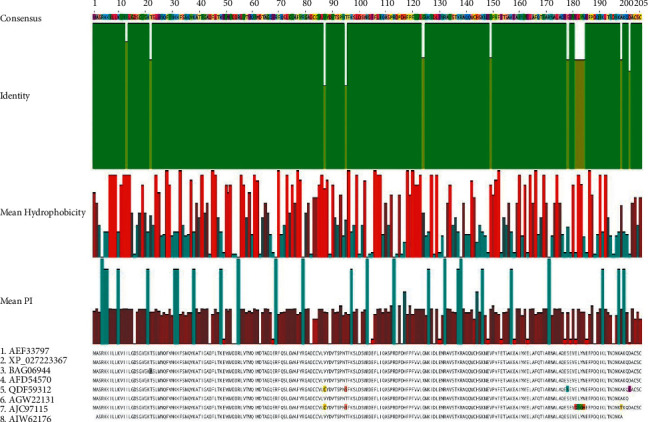
Mean hydrophobicity, isoelectric point, and identity of eight Rab7 sequences from decapod isolates after multiple sequence alignment including disagreements to consensus.

**Figure 2:**
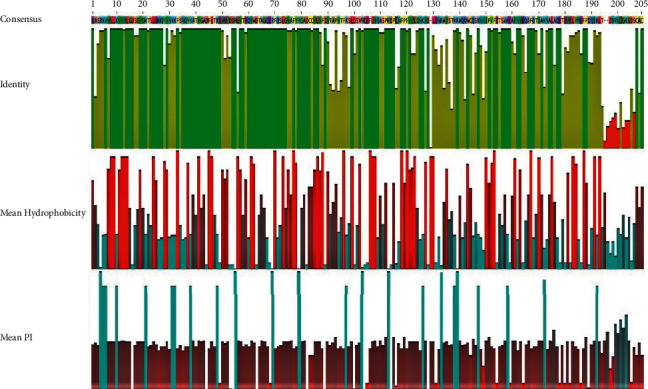
Mean hydrophobicity, isoelectric point, and identity of 100 Rab7 sequences after multiple sequence alignment.

Mean hydrophobicity, isoelectric point, and identity of crustacean Rab7 protein were graphed after multiple sequence alignment showing 13 disagreements to consensus 192 identical sites (93.7%) (Figures 1 and 2). Amino acid substitutions were observed in those 13 positions in which penaeid shrimps had in positions 13 (I ⟶ J), 22 (T ⟶ A), 124 (G ⟶ X), and 149 (V ⟶ X) while interestingly the freshwater prawn and mitten crab both had amino acid substitutions in positions 87 (V ⟶ C) and 95 (T ⟶ S) along with the other disagreements in amino acid positions 178 (S ⟶ N), 201 (D ⟶ E), 181 (E ⟶ D), 182 (L ⟶ I), 183 (Y ⟶ G), 184 (N ⟶ H) and 198 (A ⟶ T). Mean molecular weight and isoelectric point are 23.053 kDa and 5.73 respectively and a charge of −16.47 at pH 7. Mean hydrophobicity of each amino acid position is normalized and interpolated linearly to 0 to1 to prepare the graph.  Figure 2 shows the mean hydrophobicity, isoelectric point, and identity of Rab7 protein graphed after multiple sequence alignment of 100 sequences from the isolates of all groups of organisms including decapods. There are 105 amino acid identical sites (50.2%), mean molecular weight and isoelectric point of 23.269 kDa and 5.56 respectively, and a charge of −170.86 at pH 7 observed after the multiple sequence alignment in Geneious version 2020.1. Mean hydrophobicity of each amino acid position has been shown graphically after normalizing and interpolating linearly to the value of 0 to 1.

**Figure 3:**
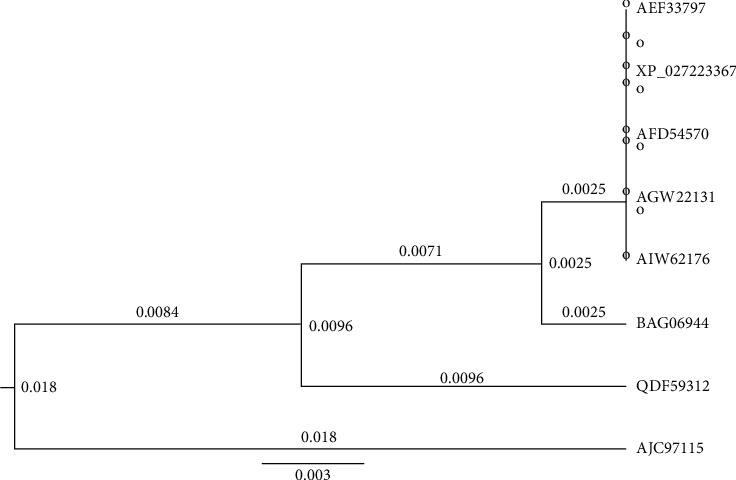
Phylogenetic tree showing evolutionary relationships of WSSV receptor protein Rab7 in *Penaeus monodon* with other closely related decapods.

**Figure 4:**
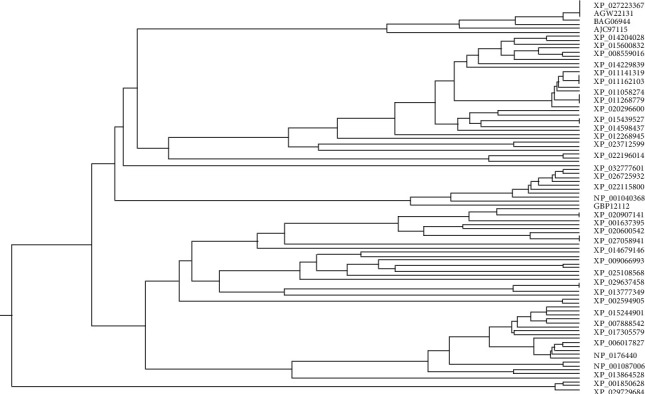
Phylogenetic tree constructed from Rab7 sequences of all groups of organisms (for a clear view all labels are not included).

**Figure 5:**
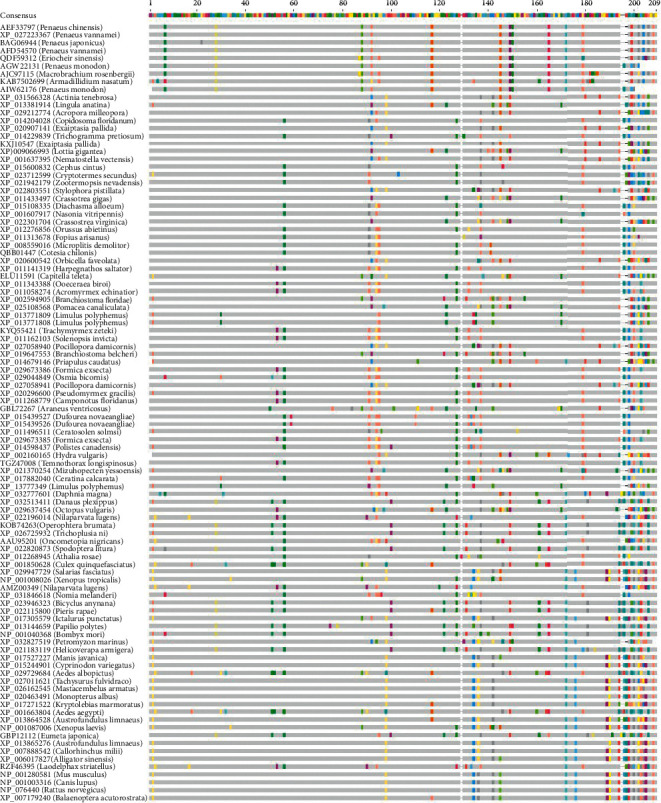
List of accession numbers of sequences with the names of organisms retrieved from NCBI showing disagreements to consensus in the colored blocks generated by geneious version 2020.1.

Figure 3 illustrates the phylogenetic tree constructed from Rab7 sequences of decapods containing 15 nodes and 8 tips. Among the decapods, *Penaeus chinensis* [GenBank: AEF33797], *Penaeus vannamei* [GenBank: XP_027223367], [GenBank: AFD54570] and *Penaeus monodon* [GenBank: AGW22131] isolates were clustered in the same group. *Penaeus japonicus* [GenBank: BAG06944], *Eriocheir sinensis* [GenBank: QDF59312], *Macrobrachium rosenbergii* [GenBank: AJC97115] and *Penaeus monodon* partial sequence [GenBank: AIW62176] of Rab7 are also not showing distant relationship. The phylogenetic tree contains 199 nodes and 100 tips in Figure 4. This tree has been drawn from sequences of 60 arthropods, 21 Chordates, 10 cnidarians, 6 molluscs, 1 priapulid, 1 annelid and 1 brachiopod that include terrestrial and aquatic organisms (marine, brackish and freshwater). Only 42 sequences are visualized here for getting a clear view (Figure 4). Few organisms listed in Figure 5 were selected (from every phylum) to observe the mutational spectrum of Rab7 (Table 2). Rab7 sequences from all phyla were compared with the *Penaeus monodon* Rab7 (GenBank: ABB70064, length: 205 aa) as the reference. Mutation analysis was considered till the 200^th^ sequence as there were variations observed while aligning to the sequences after the 200th residue from organisms of different phyla. In two cases, one in *P. japonicus* T ⟶ A (22) and another in *X. tropicalis* S ⟶ T (34), mutations have been observed in the receptor binding sites in the Rab7 sequences of all the selected organisms (Table 2). Unique mutations were meant to be mutations that were unique to the organism and common mutations were meant to the mutations that were commonly found in more than one or all organisms listed. However, common mutations were observed among organisms of the same and different phyla (Table 2).

In the decapods, the substitution varied between penaeid shrimps and others. *Macrobrachium rosenbergii*, the freshwater prawn showed similarity in amino acid substitutions with the mitten crab. Penaeid shrimps are mostly known to be affected by WSSV along with crabs of *Scylla* spp. which are cultured in coastal zones of water. Highly identical protein sequences show that this Rab7 is quite conservative in nature in the decapods. The amino acid substitutions varied in different organisms of six phyla (Figure 5) describe that there are differences in amino acids in different positions along with the length of sequences. WSSV receptor protein sequence's similarity with 60 arthropods among 100 selected according to the grade value and bit-score in Geneious version 2020.1 illustrates that there is significant similarity in the sequences of the isolates from the same phyla. Hydrophobicity and isoelectric point graphs show that there could be proper configuration in the side chain of the Rab7 protein for a steady structure.

### 3.3. Protein-Protein Interactions

**Figure 6:**
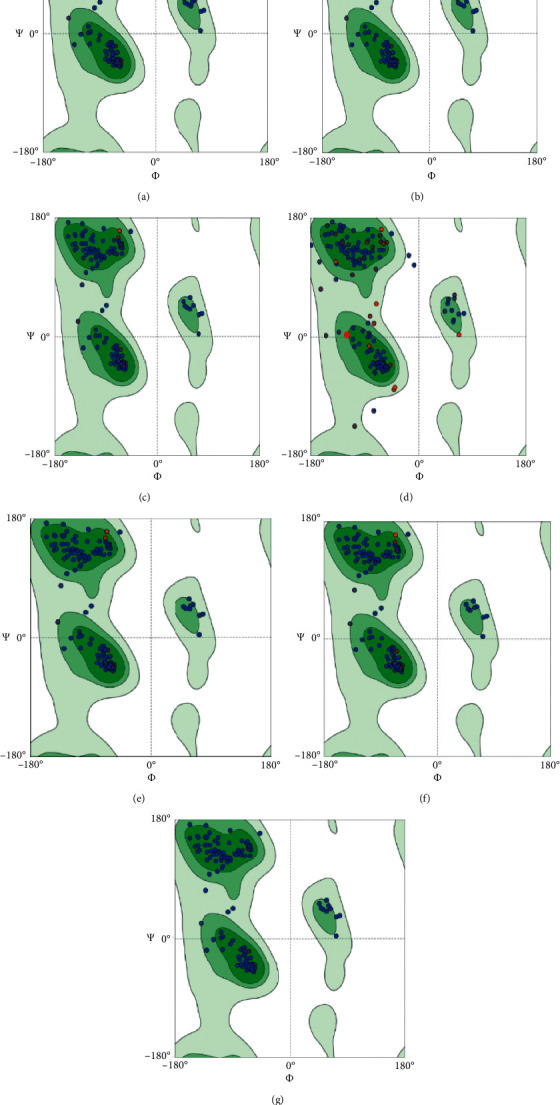
Ramachandran plots on the models from the Rab7 sequences (listed in Figure 5) of (a) *Actinia tenebrosa* (Ramachandran favored 96.70%, Ramachandran outliers 0%, rotamer outliers 1.88%), (b) *Lingula anatina* (Ramachandran favored 96.70%, Ramachandran outliers 0%, rotamer outliers 1.88%), (c) *Lottia gigantea* (Ramachandran favored 96.70%, Ramachandran outliers 0%, rotamer outliers 1.86%), (d) *Capitella teleta* (Ramachandran favored 88.95%, Ramachandran outliers 3.31%, rotamer outliers 5%), (e) *Priapulus caudatus* (Ramachandran favored 96.70%, Ramachandran outliers 0%, rotamer outliers 1.86%), (f) *Daphnia magna* (Ramachandran favored 96.70%, Ramachandran outliers 0%, rotamer outliers 1.24%), and (g) *Cyprinodon variegatus* (Ramachandran favored 96.70%, Ramachandran outliers 0%, rotamer outliers 1.85%) (MolProbity results were obtained by MolProbity version 4.4).

**Figure 7:**
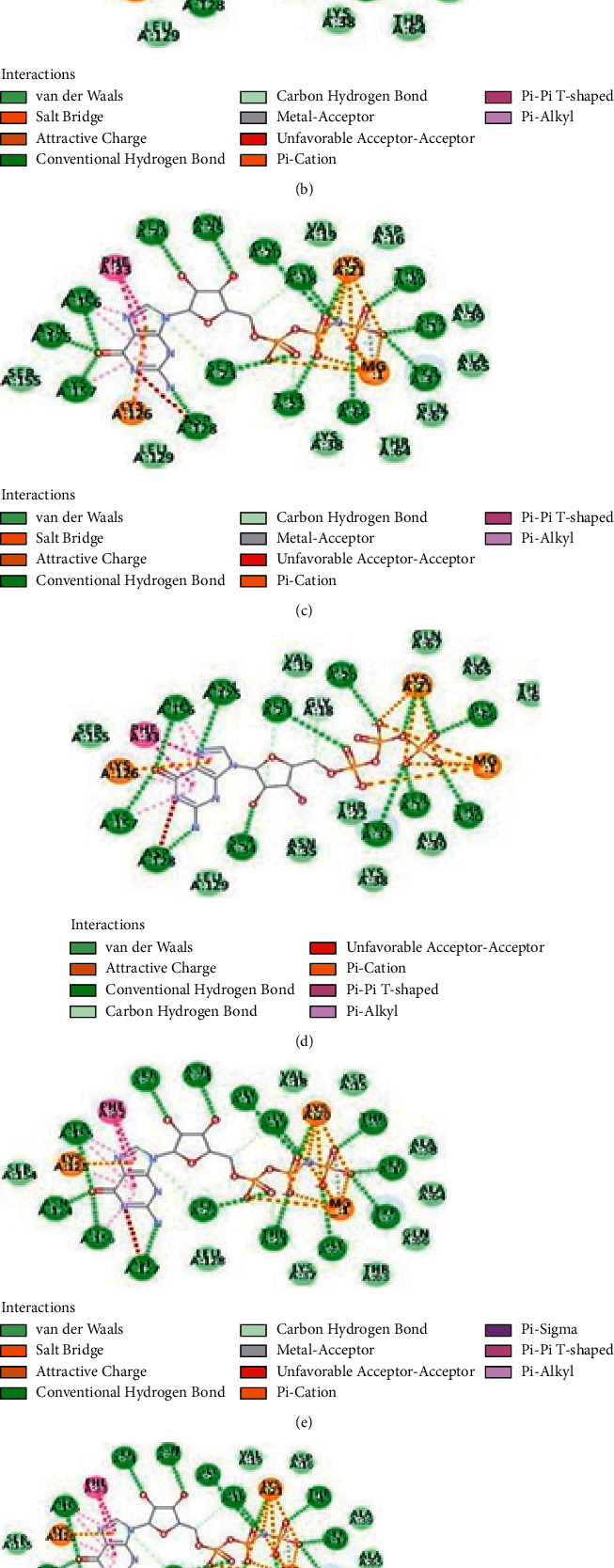
Two-dimensional view of the Rab7 interactions with ligands in the isolates of (a) *Actinia tenebrosa* (score: 19772), (b) *Lingula anatina* (score: 16980), (c) *Lottia gigantea* (score: 18822), (d) *Capitella teleta* (score: 16084), (e) *Priapulus caudatus* (score: 18714), (f) *Daphnia magna* (score: 17952), and (g) *Cyprinodon variegatus* (score: 17902) after homology modelling.

**Figure 8:**
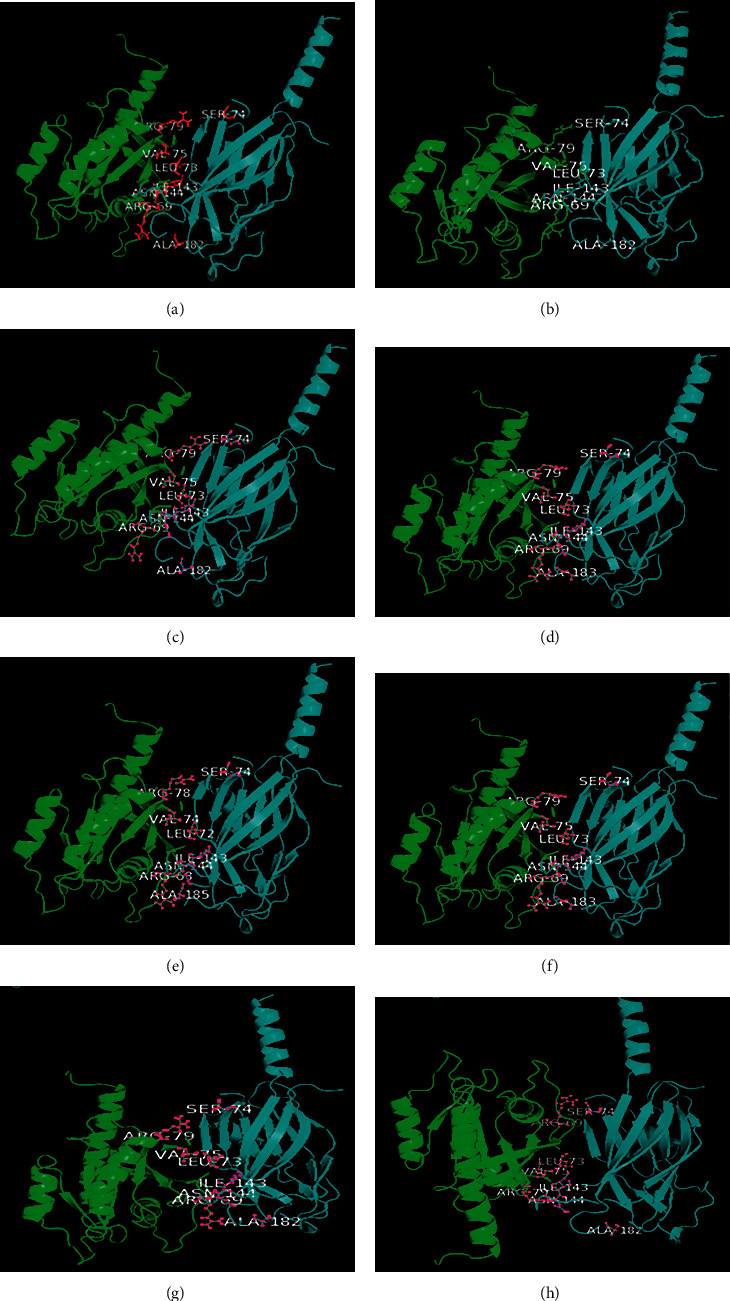
Protein-protein docking using HADDOCK 2.4 for Rab7 (left) from the isolates of (a) *Actinia tenebrosa* (b) *Lingula anatina* (c) *Lottia gigantea* (d) *Capitella teleta* (e) *Priapulus caudatus* (f) *Daphnia magna* and (g) *Cyprinodon variegatus*, and (h) a penaeid shrimp *Penaeus vannamei* with VP28 (right) of WSSV.

**Figure 9:**
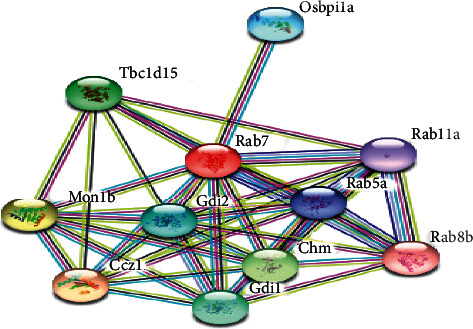
Protein-protein interaction of Rab7 generated from STRING database.

Rab7 from seven different organisms from separate phyla have been modeled and justified by constructing Ramachandran plots (Figure 6). Biologically relevant ligands of these models MG and GNP from the Swiss-Model were found to have contact with receptor Rab7 which has been visualized by Discovery Studio 4.0 (Figures 7(a)–7(g)). Receptor-ligand complex was confirmed using PatchDock Server where the score value is proportional to the energy of the binding. Phenylalanine, serine, asparagine, glycine, aspartic acid, lysine, threonine, tyrosine, and alanine of Rab7 protein were found to bind with different positions of VP28 showing different interactions (Figures 8(a)–8(g)). Rab7 sequence of *Mus musculus* [GenBank: NP_001280581] with 86.5% identity with WSSV receptor protein using STRING database (www.string-db.org) showed protein-protein interactions with Gdi1, Gdi2, Ccz1, Chm, Mon1b, Tbc1d15, Osbpl1a, and other proteins from Rab family-Rab5a, Rab8b and Rab11a (Figure 9) [23].

Interactions of proteins are natural associations of greater specificity set concerning two or additional protein molecules for biochemical events guided by connections that incorporate hydrogen bonding, electrostatic forces, and hydrophobic effect [24]. It is very well known that structures of proteins are more conserved than sequences of protein and DNA amongst homologs though noticeable levels of sequence similarity usually extrapolate substantial resemblance in structure [25]. Figures 6(a)–6(g) shows the Ramachandran plot results which indicate that the models generated using SWISS-MODEL were suitable [26]. Active residues of Rab7 Arginine 69, Leucine 73, Valine 75 and Arginine 79 in the selected organisms and VP28's Serine 74, Isoleucine 143, Asparagine 144, and Alanine 182 were placed in the binding interface (Figure 8). In Figures 8(a)–8(g), Arginine at position 69 of Rab7 is placed at the below part of the complex's binding interface while Arginine at position 79 is placed at the upper part unlike in Figure 8(h). All the other active residues of Rab7 are found placed in the middle of Figures 8(a)–8(g)) in a slightly different manner than in Figure 8(h) which is of a penaeid shrimp.  Table 3 contains the protein-protein docking information derived from the number of combinations in the input molecules. HADDOCK 2.4 produces a number of clusters for the development of the complex. On the basis of the energy structures and *Z*-score, the best cluster has been considered. *Z*-score showed the number of standard deviations from the average the cluster is placed. In these clusters generated by HADDOCK 2.4, the more negative one is the better one. There is an opinion that PmRab7 may not be responsible for the entry of the virus into the host, but it might bind to the viral protein at a later point [27]. Researchers opined that there could be possibility of formation of a multiprotein complex of several structural proteins of WSSV prior to attaching to the host protein, and a minimum of five complexes of structural proteins had been mentioned for binding to the receptor [28–30]. However, the current *in-silico* study has observed the points where VP28 of WSSV can bind to the models of Rab7 showing the possibility of their affinity to binding to Rab7 of organisms living in the marine or brackish water. Among the 100 sequences of isolates mentioned in this study, 7 sequences of Rab7 were selected from different phyla to observe the binding between Rab7 and VP28 in *Actinia tenebrosa*, *Lingula anatina*, *Lottia gigantea*, *Capitella teleta*, *Priapulus caudatus*, *Daphnia magna*, and *Cyprinodon variegatus*. There has been predicted significant binding between these proteins of hosts and the pathogen. Only two mutations observed in the receptor binding sites of these selected organisms' Rab7. It can be assumed from the mutational spectrum analysis that there could be higher possibility of protein-protein binding. The long-conserved domains in Rab7 sequences with identical residues have the similar interaction interfaces. However, Hameed et al. found that WSSV could not be infected with *Artemia* at its developing phase by immersion trial, which was checked by PCR negative finding pointing out *Artemia* may well not be a carrier, yet they recommended further studies [31]. And on the other hand, rotifers were found to be experimentally infected and the confirmation was done by conventional PCR [32]. Rotifers in natural samples were found WSSV positive collected from shrimp ponds and the rotifer resting eggs by PCR stating rotifers as the vectors or carriers of WSSV responsible for transmitting to shrimps or crabs [33]. In a different study, WSSV was found in the Pacific oyster *Crassostrea gigas* but its susceptibility to infection could not be determined and its function as a carrier was not established [34]. Highest prevalence of WSSV was found in taxa of copepods, brachyurous, and bivalves while observing the prevalence of WSSV in zooplankton samples [35], and the researchers of the study also stated that 12 taxa could be high-risk vectors of WSSV. As Rab7 was found in different groups of organisms and has been docked in this study for protein-protein binding with VP28 of WSSV showing a possibility of binding with active sites of Rab7 of these organisms like in PmRab7, there could be such binding in few other organisms in which WSSV may be able to develop infection or which may be vectors leading to transmission into shrimps and crabs those live in salt water. In this study, the binding sites were similar in all the other organisms other than *P. vannamei*. The simulation and docking of PmRab7 [36] revealed Arginine 69, Leucine 73, Valine 75, Arginine 79, and Alanine 198 as the active sites. Rab7-VP28 complex showed similar interface and binding affinity in our studied *P. vannamei* and *P. monodon* [36] while in other selected organisms Rab7 is placed in the way that Arginine 79 was close to Serine 74 of VP28 and Arginine 69 was placed below and close to Asparagine 144. Leucine 73 and Valine 75 were shown to have closely placed with Isoleucine 143 in all organisms including *P. vannamei*. Arginine, present whether in position 69 or 79 of Rab7, may be able to bind to Serine at position 74 like in penaeid shrimps as these were placed closely in all organisms. Alanine 198 was absent in the chosen sequences for docking in this study like *P. monodon* which might have changed slightly the placement of Rab7 in the complexes formed. Since the active residues are present in the interaction interface and WSSV is present in a list of marine organisms other than penaeid shrimps, there is a possibility of binding of Rab7 and VP28 in marine organisms. Although there are many other factors involved in host-virus relationship, the presence of Rab7 in marine water organisms may play a significant role in the existence of such a relationship.

### 3.4. Final Remarks

Rab7 protein of penaeid shrimp is involved in binding an envelope protein of WSSV known as VP28. Focusing on the statement, Rab7 sequences were retrieved from NCBI, and alignment of 100 sequences was done along with a separate alignment of sequences of 8 crustaceans. Isoelectric points and hydrophobicity points might play a very important role in binding to the viral envelop protein or protein complex. It was observed that the Rab7 identical sequences varied from 86.5 to 100% in all organisms while it varied from 94.6 to 100% in decapods that including shrimps and crabs. We suggest Rab7 expressing the gene in the marine organisms can be a marker showing the possibility of such a protein-protein binding in the organisms that could host WSSV and transmit the virus to shrimps and crabs of economic importance. Mutational analysis and the anticipated possibility of VP28-Rab7 binding in different organisms with similar interaction interfaces rise the possibility that there could be several hosts of WSSV which might act as carriers and can be responsible for the spread of WSSV in commercially important crustacean farms. Binding assays *in vitro* can demonstrate the binding of VP28 to Rab7 present in different marine organisms' host cells. Moreover, the structural basis for protein recognition and key intermolecular interactions through crystallization and CyaA translocation assay can provide further insights.

## Figures and Tables

**Table 1:** Annotation results of Rab7 protein sequences using geneious prime version 2020.1.

Name	Type	Minimum	Maximum	Length	Interval
Rab subfamily	Site	171	179	9	1
G5 box	Site	155	157	3	1
G4 box	Site	125	128	4	1
Rab subfamily	Site	117	122	6	1
Rab family	Site	86	91	6	1
Rab family	Site	77	81	5	1
Rab family	Site	69	74	6	1
Switch II region	Site	66	78	12	2
G3 box	Site	63	66	4	1
Rab family	Site	58	62	5	1
Putative effector binding site	Site	41	173	15	9
Rab family	Site	41	45	5	1
G2 box	Site	40	40	1	1
Other	Site	38	58	11	4
Switch I region	Site	33	46	10	2
Rab subfamily	Site	23	39	16	2
Other	Site	17	157	17	8
GDI Interaction site	Site	17	79	13	8
G1 box	Site	15	22	8	1
Rab7	Region	9	179	171	1
RAB	Region	9	176	168	1
Rab subfamily	Site	9	10	2	1
Rab7 CDS	CDS	1	205	205	1
WSSV receptor	Protein	1	205	205	1

**Table 2:** Phyla-wise mutations in Rab7 sequences of different phyla.

Phylum	Unique mutation	Common mutation
Arthropoda		T ⟶ S (95), E ⟶ D (181)
*Penaeus monodon* (AGW22131)		
*Penaeus vannamei* (AFD54570)		
*Macrobrachium rosenbergii* (AJC97115)	V ⟶ C (87), L ⟶ I (182), Y ⟶ G (183), N ⟶ H (184)	
*Penaeus japonicus* (BAG06944)	T ⟶ A (22)	
*Daphnia magna* (XP_032777601)	K ⟶ R (5), F ⟶ Y (28), K ⟶ R (31), Y ⟶ F (88), N ⟶ T (94), S ⟶ D (98), D ⟶ E (116), H ⟶ N (117), I ⟶ V (127), S ⟶ A (145), E ⟶ D (148), V ⟶ I (149), A ⟶ G (159), L ⟶ Q (164), S ⟶ T (178), E ⟶ Q (179), D ⟶ E (188), N ⟶ G (195), N ⟶ Q (197)	

Chordata		A ⟶ T (2), I ⟶ V (7), F ⟶ Y (28), Y ⟶ F (88), S ⟶ A (92), S ⟶ T (98), D ⟶ E (116), H ⟶ N (117), A ⟶ Q (133), S ⟶ T (135), Q ⟶ A (141), H ⟶ Q (144), E ⟶ N (148), V ⟶ I (149), L ⟶ Q (164), A ⟶ K (175), S ⟶ T (178), D ⟶ E (188), Q ⟶ P (189), T ⟶ D (193), N ⟶ R (194), D ⟶ N (195), N ⟶ D (196), K ⟶ R (197), Q ⟶ P (200)
* Cyprinodon variegatus* (XP_015244901)		
* Monopteros albus* (XP_020463491)		
* Tachysurus fulvidraco* (XP_027011621		
* Xenopus tropicalis* (NP_001008026)	S ⟶ T (34), I ⟶ V (127), Q ⟶ V (141), N ⟶ K (194), Q ⟶ A (200)	
* Mastacembelus armatus* (XP_026162545)	Q ⟶ T (200)	
Annelida		

* Capitella teleta* (ELU11591)	A ⟶ T (2), I ⟶ V (7), F ⟶ Y (28), S ⟶ V (92), S ⟶ T (98), D ⟶ E (116), H ⟶ N (117), L ⟶ I (123), K ⟶ R (137), H ⟶ Q (144), S ⟶ T (145), N ⟶ G (147), E ⟶ D (148), V ⟶ I (149), L ⟶ Q (164), I ⟶ V (169), R ⟶ K (171), S ⟶ T (178), T ⟶ S (193), N ⟶ Q (196), K ⟶ N (197), A ⟶ K (198), K ⟶ P (199), Q ⟶ K (200)	

Cnideria		I ⟶ V (7), F ⟶ Y (28), Y ⟶ F (88), S ⟶ T (98), D ⟶ E (116), H ⟶ N (117), T ⟶ A (136), Q ⟶ A (141), E ⟶ D (148), V ⟶ I (149), L ⟶ Q (164), R ⟶ K (171), S ⟶ T (178), E ⟶ D (179), E ⟶ D (185), T ⟶ S (193), N ⟶ G (194), N ⟶ S (196), A ⟶ P (198), A ⟶ Q (198), Q ⟶ S (200)
* Actinia tenebrosa* (XP_031566328)		
* Hydra vulgaris* (XP_002160165)	S ⟶ A (92), T ⟶ S (95), I ⟶ V (127), A ⟶ G (159), L ⟶ H (164), N ⟶ K (172), S ⟶ A (178), T ⟶ N (193), N ⟶ P (194), K ⟶ R (199)	
* Orbicella faveolata* (XP_020600542)	N ⟶ S (94), S ⟶ N (135), Q ⟶ S (141)	
* Exaiptasia pallida* (XP_020907141)	E ⟶ N (148), D ⟶ E (195), Q ⟶ A (200)	
* Stylophora pistillata* (XP_022803551)	N ⟶ T (94), A ⟶ V (133), S ⟶ M (135), K ⟶ R (191)	

Mollusca		I ⟶ V (7), F ⟶ Y (28), S ⟶ M (92), K ⟶ R (97), D ⟶ E (116), H ⟶ N (117), L ⟶ I (123), S ⟶ T (135), T ⟶ A (136), Q ⟶ S (141), Q ⟶ G (141), S ⟶ T (145), N ⟶ G (147), V ⟶ I (149), L ⟶ Q (164), I ⟶ V (169), R ⟶ K (171), S ⟶ T (178), E ⟶ D (179), L ⟶ I (192), T ⟶ S (193), N ⟶ Q (196), K ⟶ N (197), A ⟶ K (198), K ⟶ P (199), Q ⟶ K (200), Q ⟶ R (200)
* Lottia gigantea* (XP_009066993)	F ⟶ Y (98), A ⟶ S (139), N ⟶ G (196)	
* Mizuhopecten yessoensis* (XP_021370254)	A ⟶ S (2), H ⟶ N (144), S ⟶ N (178), N ⟶ Q (196)	
* Crassostrea virginica* (XP_022301704)	H ⟶ T (144), N ⟶ T (196), K ⟶ Q (199), Q ⟶ D (200)	
* Pomacea canaliculata* (XP_025108568)	H ⟶ S (144), K ⟶ T (197), A ⟶ N (198)	
* Octopus vulgaris* (XP_029637458)	D ⟶ E (53), S ⟶ A (92), P ⟶ Q (93), N ⟶ T (94), N ⟶ S (194)	
Brachiopoda		
* Lingula anatina* (XP_013381914)	A ⟶ S (2), I ⟶ V, F ⟶ Y (28), Y ⟶ F (88), S ⟶ M (92), S ⟶ T (98), D ⟶ E (116), L ⟶ I (123), Q ⟶ G (141), N ⟶ G (147), E ⟶ D (148), V ⟶ I (149), L ⟶ Q (164), I ⟶ V (169), R ⟶ K (171), S ⟶ T (178), N ⟶ A (197), K ⟶ N (198), A ⟶ K (199)	

Priapulida		
* Priapulus caudatus* (XP_014679146)	A ⟶ S (2), I ⟶ V (7), F ⟶ Y (28), Y ⟶ F (88), S ⟶ Q (92), S ⟶ G (111), H ⟶ N (117), Q ⟶ G (141), E ⟶ D (148), V ⟶ I (149), A ⟶ S (159), L ⟶ Q (164), S ⟶ T (178), E ⟶ D (179), E ⟶ D (185), N ⟶ G (194), D ⟶ E (195), N ⟶ T (196), A ⟶ P (198), K ⟶ P (199), Q ⟶ S (200)	

**Table 3:** Docking information of Rab7 protein sequences of different species.

Species	Score	RMSD from the overall lowest energy structure	Van der Waals energy	Electrostatic energy	Desolvation energy	Restraints violation energy	Buried surface area	*Z*-score
*Actinia tenebrosa*	−79.3 ± 7.3	20.8 ± 0.3	−46.5 ± 3.5	−167.2 ± 58.2	−4.8 ± 5.7	53.5 ± 17.9	1510.8 ± 20.9	−2.0

*Lingula anatina*	−72.3 ± 2.2	15.7 ± 0.2	−45.5 ± 2.7	−123.6 ± 9.3	−9.0 ± 2.2	70.2 ± 32.5	1467.0 ± 32.8	−1.3

*Lottia gigantea*	−76.3 ± 2.3	7.6 ± 0.4	−48.4 ± 5.6	−119.2 ± 35.4	−10.7 ± 1.1	66.8 ± 33.1	1482.7 ± 52.2	−1.7

*Capitella teleta*	−76.2 ± 3.7	1.0 ± 0.6	−37.4 ± 1.4	−237.5 ± 6.1	3.7 ± 2.8	49.6 ± 9.3	1366 ± 56.9	−1.5

*Priapulus caudatus*	−71.6 ± 1.7	4.0 ± 0.5	−44.6 ± 4.5	−117.4 ± 16.7	−8.0 ± 1.2	44.8 ± 29.8	1488.6 ± 104.2	−1.8

*Daphnia magna*	−74.1 ± 3.5	21.0 ± 0.3	−48.3 ± 3.9	−122.4 ± 34.6	−8.3 ± 1.4	70.1 ± 25.4	1470.2 ± 54.0	−1.7

*Cyprinodon variegatus*	−80.1 ± 3.5	7.6 ± 0.8	−45.6 ± 4.1	−160.9 ± 30.3	−9.0 ± 2.1	66.6 ± 24.6	1485.3 ± 40.4	−1.6

*Penaeus vannamei*	−84.9 ± 24.8	1.1 ± 0.8	−36.8 ± 3.9	−263.0 ± 123.4	0.1 ± 4.3	43.5 ± 25.1	1424.0 ± 129.8	−1.9

## Data Availability

All data are available upon request.
